# *t*-3-Benzyl-*r*-2,*c*-6-bis­(4-methoxy­phen­yl)piperidin-4-one oxime

**DOI:** 10.1107/S1600536808016449

**Published:** 2008-06-07

**Authors:** J. Jayabharathi, A. Thangamani, S. Balamurugan, A. Thiruvalluvar, A. Linden

**Affiliations:** a Department of Chemistry, Annamalai University, Annamalai Nagar 608 002, Tamil Nadu, India; b PG Research Department of Physics, Rajah Serfoji Government College (Autonomous), Thanjavur 613 005, Tamil Nadu, India; c Institute of Organic Chemistry, University of Zürich, Winterthurerstrasse 190, CH-8057 Zürich, Switzerland

## Abstract

In the title mol­ecule, C_26_H_28_N_2_O_3_, the piperidine ring adopts a chair conformation. The two methoxy­phenyl groups attached to the piperidine ring at positions 2 and 6 have equatorial orientations, and make a dihedral angle of 80.72 (15)°. The benzyl group at position 3 has an equatorial orientation. The oxime group at position 4 has a bi­sectional orientation. The ring of the benzyl group makes dihedral angles of 64.71 (16) and 84.79 (17)° with the two benzene rings. Mol­ecules are linked by inter­molecular N—H⋯O, O—H⋯N and C—H⋯O hydrogen bonds, and C—H⋯π inter­actions. There is also a C—H⋯O intra­molecular inter­action.

## Related literature

For related literature, see: Jayabharathi *et al.* (2007 ▶); Thiruvalluvar *et al.* (2007 ▶).
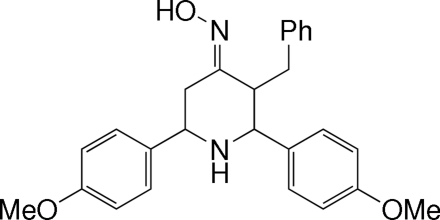


## Experimental

### 

#### Crystal data

C_26_H_28_N_2_O_3_
*M* *_r_* = 416.50
Orthorhombic, 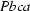

*a* = 10.2472 (4) Å
*b* = 11.2723 (4) Å
*c* = 38.4188 (15) Å
*V* = 4437.7 (3) Å^3^
*Z* = 8
Mo *K*α radiation
μ = 0.08 mm^−1^
*T* = 160 (1) K
0.25 × 0.13 × 0.10 mm

#### Data collection

Nonius KappaCCD area-detector diffractometer
Absorption correction: none
36542 measured reflections
3910 independent reflections
2305 reflections with *I* > 2σ(*I*)
*R*_int_ = 0.092

#### Refinement

*R*[*F* ^2^ > 2σ(*F* ^2^)] = 0.070
*wR*(*F* ^2^) = 0.211
*S* = 1.08
3910 reflections
286 parameters
H atoms treated by a mixture of independent and constrained refinement
Δρ_max_ = 0.88 e Å^−3^
Δρ_min_ = −0.75 e Å^−3^

### 

Data collection: *COLLECT* (Nonius, 2000 ▶); cell refinement: *DENZO-SMN* (Otwinowski & Minor, 1997 ▶); data reduction: *DENZO-SMN* and *SCALEPACK* (Otwinowski & Minor, 1997 ▶); program(s) used to solve structure: *SHELXS97* (Sheldrick, 2008 ▶); program(s) used to refine structure: *SHELXL97* (Sheldrick, 2008 ▶); molecular graphics: *ORTEP-3* (Farrugia, 1997 ▶); software used to prepare material for publication: *PLATON* (Spek, 2003 ▶).

## Supplementary Material

Crystal structure: contains datablocks global, I. DOI: 10.1107/S1600536808016449/wn2266sup1.cif

Structure factors: contains datablocks I. DOI: 10.1107/S1600536808016449/wn2266Isup2.hkl

Additional supplementary materials:  crystallographic information; 3D view; checkCIF report

## Figures and Tables

**Table 1:** Hydrogen-bond geometry (Å, °) *Cg*1 and *Cg*2 are the centroids of rings C31–C36 and C21–C26, respectively.

*D*—H⋯*A*	*D*—H	H⋯*A*	*D*⋯*A*	*D*—H⋯*A*
N1—H1⋯O6^i^	0.97 (3)	2.52 (3)	3.348 (3)	144 (2)
O4—H4⋯N1^ii^	0.84	2.01	2.818 (3)	160
C5—H5*A*⋯O4	0.99	2.27	2.705 (4)	105
C65—H65⋯O4^iii^	0.95	2.43	3.334 (4)	158
C12—H12*B*⋯*Cg*1^iv^	0.98	2.89	3.314 (4)	107
C16—H16*A*⋯*Cg*2^i^	0.98	2.65	3.598 (4)	162

Symmetry codes: (i) 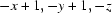; (ii) 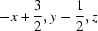; (iii) 
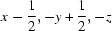
; (iv) 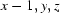.

**Fig. 1.**
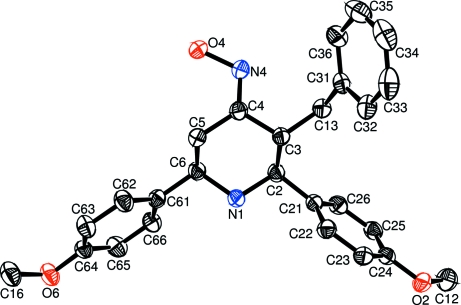
The molecular structure of the title compound, showing the atom-numbering scheme and displacement ellipsoids drawn at the 50% probability level. H atoms have been omitted for clarity.

**Fig. 2.**
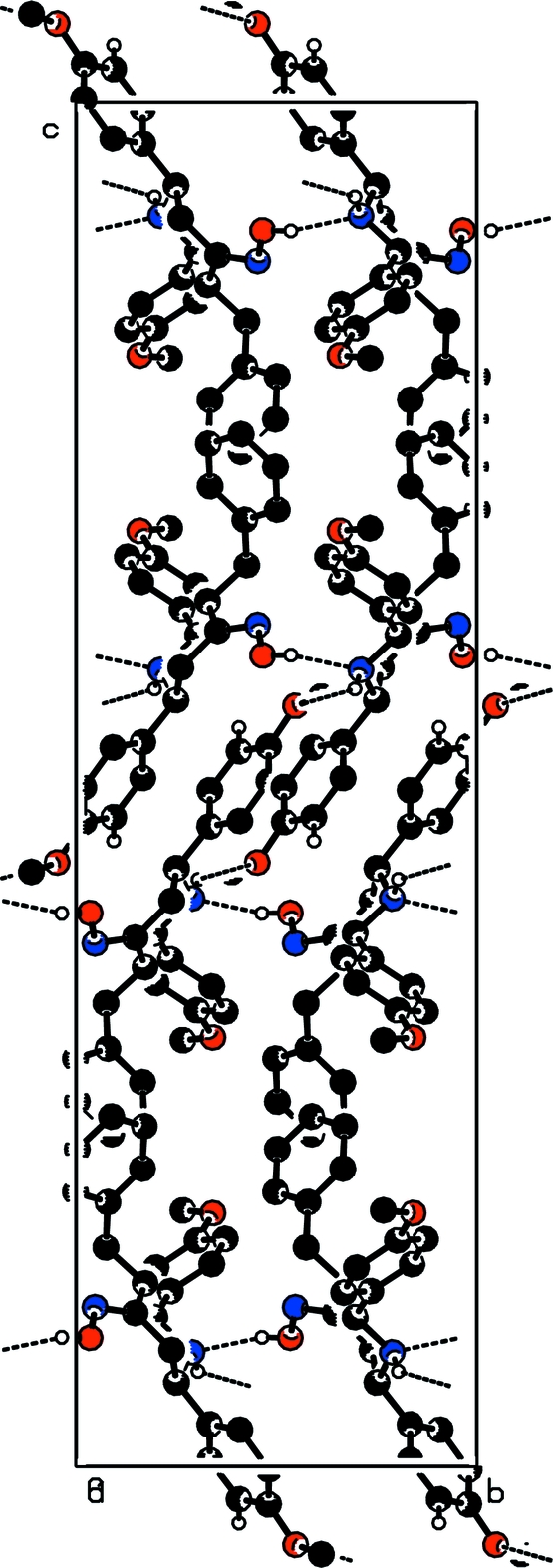
The molecular packing of the title compound, viewed down the a axis. Dashed lines indicate hydrogen bonds. H atoms not involved in hydrogen bonding have been omitted.

## supplementary crystallographic information

### Comment

In a wide research program toward new and efficient antimicrobial agents, a series of t3-benzyl-r2,c6-diarylpiperidin-4-ones have been synthesized and tested for their *in vitro* antibacterial and antifungal activities (Jayabharathi *et al.*, 2007). Thiruvalluvar *et al.* (2007) have reported the crystal structure of t3-benzyl-1-formyl-r2,c-6-diphenylpiperidin-4-one, in which the piperidine ring is in a distorted boat form.

In the title compound, (Fig. 1), the piperidine ring adopts a chair conformation. The two methoxyphenyl groups attached to the piperidine ring at positions 2 and 6 have equatorial orientations, and make a dihedral angle of 80.72 (15)°. The benzyl group at position 3 has an equatorial orientation. The oxime group at position 4 has a bisectional orientation. The phenyl ring of the benzyl group makes a dihedral angle of 64.71 (16)° with the benzene ring at C2, and 84.79 (17)° with the benzene ring at C6. Molecules are linked by intermolecular N1—H1···O6, O4—H4···N1 and C65—H65···O4 hydrogen bonds (Fig. 2). There are C12—H12B···π (-1 + *x*, *y*, *z*) interactions involving the phenyl ring at C13 and C16—H16A···π (1 - *x*, 1 - *y*, -*z*) interactions involving the benzene ring at C2. There is also a C5—H5A···O4 intramolecular interaction.

### Experimental

The title compound was prepared according to the literature procedure (Jayabharathi *et al.*, 2007). t3-Benzyl-r2,c6-bis(*p*-methoxyphenyl)piperidin-4-one (ca 0.05 mol, 20 g) and sodium acetate trihydrate (0.15 mol, 20.41 g) were dissolved in boiling ethanol and hydroxylamine hydrochloride (0.06 mol, 4.17 g) was added. The mixture was heated to 313 K and stirred for 3-4 h. It was allowed to stand overnight and then poured into crushed ice. The separated solid was filtered off and recrystallized from ethanol. The yield of the isolated pure product was 16 g (80%).

### Refinement

The H atom bonded to N1 was located in a difference Fourier map and refined isotropically to an N—H bond length of 0.97 (3) Å. The remaining H atoms were positioned geometrically and allowed to ride on their parent atoms, with C—H = 0.95–1.00 Å and O—H = 0.84 Å; U_iso_(H) = xU_eq_(carrier atom), where x = 1.5 for methyl and O, 1.2 for all others.

### Crystal data

**Table:**

C_26_H_28_N_2_O_3_	*D*_x_ = 1.247 Mg m^−^^3^
*M**_r_* = 416.50	Melting point: 460 K
Orthorhombic, *P**b**c**a*	Mo *K*α radiation λ = 0.71073 Å
Hall symbol: -P 2ac 2ab	Cell parameters from 4402 reflections
*a* = 10.2472 (4) Å	θ = 2.0–25.0º
*b* = 11.2723 (4) Å	µ = 0.08 mm^−^^1^
*c* = 38.4188 (15) Å	*T* = 160 (1) K
*V* = 4437.7 (3) Å^3^	Tablet, colourless
*Z* = 8	0.25 × 0.13 × 0.10 mm
*F*_000_ = 1776	

### Data collection

**Table:**

Nonius KappaCCD area-detector diffractometer	3910 independent reflections
Radiation source: Nonius FR590 sealed tube generator	2305 reflections with *I* > 2σ(*I*)
Monochromator: horizontally mounted graphite crystal	*R*_int_ = 0.092
Detector resolution: 9 pixels mm^-1^	θ_max_ = 25.0º
*T* = 160(1) K	θ_min_ = 3.1º
φ and ω scans with κ offsets	*h* = −12→12
Absorption correction: none	*k* = −13→13
36542 measured reflections	*l* = −45→45

### Refinement

**Table:**

Refinement on *F*^2^	Hydrogen site location: inferred from neighbouring sites
Least-squares matrix: full	H atoms treated by a mixture of independent and constrained refinement
*R*[*F*^2^ > 2σ(*F*^2^)] = 0.070	*w* = 1/[σ^2^(*F*_o_^2^) + (0.1121*P*)^2^] where *P* = (*F*_o_^2^ + 2*F*_c_^2^)/3
*wR*(*F*^2^) = 0.211	(Δ/σ)_max_ < 0.001
*S* = 1.08	Δρ_max_ = 0.88 e Å^−^^3^
3910 reflections	Δρ_min_ = −0.75 e Å^−^^3^
286 parameters	Extinction correction: SHELXL97 (Sheldrick, 2008), Fc^*^=kFc[1+0.001xFc^2^λ^3^/sin(2θ)]^-1/4^
Primary atom site location: structure-invariant direct methods	Extinction coefficient: 0.036 (3)
Secondary atom site location: difference Fourier map	

### Special details

**Table:**

Experimental. Solvent used: Ethanol Cooling Device: Oxford Cryosystems Cryostream 700 Crystal mount: glued on a glass fibre Mosaicity (°.): 0.618 (1) Frames collected: 629 Seconds exposure per frame: 35 Degrees rotation per frame: 1.0 Crystal-Detector distance (mm): 57.3
Geometry. Bond distances, angles *etc*. have been calculated using the rounded fractional coordinates. All su's are estimated from the variances of the (full) variance-covariance matrix. The cell e.s.d.'s are taken into account in the estimation of distances, angles and torsion angles
Refinement. Refinement of *F*^2^ against ALL reflections. The weighted *R*-factor *wR* and goodness of fit *S* are based on *F*^2^, conventional *R*-factors *R* are based on *F*, with *F* set to zero for negative *F*^2^. The threshold expression of *F*^2^ > σ(*F*^2^) is used only for calculating *R*-factors(gt) *etc*. and is not relevant to the choice of reflections for refinement. *R*-factors based on *F*^2^ are statistically about twice as large as those based on *F*, and *R*- factors based on ALL data will be even larger.

### Fractional atomic coordinates and isotropic or equivalent isotropic displacement parameters (Å^2^)

**Table:**

	*x*	*y*	*z*	*U*_iso_*/*U*_eq_	
O2	0.0668 (2)	0.34156 (19)	0.18566 (6)	0.0420 (8)	
O4	0.9150 (2)	0.03452 (19)	0.09433 (6)	0.0386 (8)	
O6	0.7048 (2)	0.5478 (2)	−0.05739 (5)	0.0443 (9)	
N1	0.5498 (3)	0.2892 (2)	0.08373 (6)	0.0311 (9)	
N4	0.8055 (2)	0.0468 (2)	0.11664 (6)	0.0335 (9)	
C2	0.5128 (3)	0.1965 (3)	0.10941 (8)	0.0322 (11)	
C3	0.6286 (3)	0.1719 (3)	0.13407 (8)	0.0301 (10)	
C4	0.7480 (3)	0.1470 (3)	0.11248 (8)	0.0315 (11)	
C5	0.7802 (3)	0.2386 (3)	0.08540 (8)	0.0354 (11)	
C6	0.6613 (3)	0.2506 (3)	0.06163 (8)	0.0332 (11)	
C12	−0.0455 (3)	0.2668 (3)	0.18763 (10)	0.0550 (16)	
C13	0.5957 (3)	0.0740 (3)	0.16052 (8)	0.0356 (11)	
C16	0.8212 (3)	0.6119 (3)	−0.06432 (9)	0.0533 (14)	
C21	0.3912 (3)	0.2353 (3)	0.12845 (7)	0.0292 (10)	
C22	0.3900 (3)	0.3387 (3)	0.14823 (8)	0.0339 (11)	
C23	0.2808 (3)	0.3721 (3)	0.16668 (8)	0.0355 (11)	
C24	0.1683 (3)	0.3020 (3)	0.16566 (8)	0.0338 (11)	
C25	0.1667 (3)	0.1996 (3)	0.14565 (8)	0.0367 (12)	
C26	0.2785 (3)	0.1679 (3)	0.12733 (8)	0.0357 (11)	
C31	0.6756 (3)	0.0789 (3)	0.19370 (8)	0.0341 (11)	
C32	0.6473 (3)	0.1655 (3)	0.21837 (8)	0.0433 (12)	
C33	0.7156 (4)	0.1699 (4)	0.24939 (10)	0.0553 (16)	
C34	0.8112 (4)	0.0882 (4)	0.25647 (10)	0.0570 (16)	
C35	0.8412 (4)	0.0025 (3)	0.23256 (10)	0.0537 (14)	
C36	0.7748 (3)	−0.0020 (3)	0.20113 (9)	0.0433 (12)	
C61	0.6775 (3)	0.3337 (3)	0.03083 (8)	0.0323 (11)	
C62	0.7835 (3)	0.4086 (3)	0.02694 (8)	0.0413 (12)	
C63	0.7969 (3)	0.4812 (3)	−0.00223 (8)	0.0413 (12)	
C64	0.7021 (3)	0.4801 (3)	−0.02775 (8)	0.0350 (11)	
C65	0.5951 (3)	0.4067 (3)	−0.02416 (8)	0.0420 (12)	
C66	0.5834 (3)	0.3338 (3)	0.00450 (8)	0.0377 (12)	
H1	0.473 (3)	0.304 (2)	0.0698 (8)	0.030 (8)*	
H2	0.49258	0.12179	0.09644	0.0385*	
H3	0.64551	0.24632	0.14749	0.0362*	
H4	0.94084	−0.03620	0.09464	0.0578*	
H5A	0.85738	0.21356	0.07175	0.0422*	
H5B	0.79998	0.31560	0.09663	0.0422*	
H6	0.64031	0.17003	0.05231	0.0397*	
H12A	−0.11135	0.30392	0.20258	0.0825*	
H12B	−0.02061	0.18972	0.19741	0.0825*	
H12C	−0.08165	0.25535	0.16425	0.0825*	
H13A	0.50211	0.08010	0.16667	0.0426*	
H13B	0.60941	−0.00410	0.14934	0.0426*	
H16A	0.81134	0.65636	−0.08607	0.0794*	
H16B	0.89431	0.55628	−0.06650	0.0794*	
H16C	0.83840	0.66712	−0.04517	0.0794*	
H22	0.46583	0.38715	0.14903	0.0407*	
H23	0.28182	0.44291	0.18010	0.0423*	
H25	0.09043	0.15182	0.14448	0.0437*	
H26	0.27738	0.09781	0.11362	0.0430*	
H32	0.58053	0.22197	0.21388	0.0523*	
H33	0.69611	0.23001	0.26593	0.0666*	
H34	0.85656	0.09114	0.27801	0.0684*	
H35	0.90752	−0.05401	0.23749	0.0643*	
H36	0.79716	−0.06079	0.18445	0.0518*	
H62	0.84854	0.41066	0.04454	0.0494*	
H63	0.87098	0.53122	−0.00453	0.0494*	
H65	0.52922	0.40631	−0.04155	0.0504*	
H66	0.50995	0.28279	0.00639	0.0456*	

### Atomic displacement parameters (Å^2^)

**Table:**

	*U*^11^	*U*^22^	*U*^33^	*U*^12^	*U*^13^	*U*^23^
O2	0.0306 (14)	0.0431 (15)	0.0524 (15)	0.0035 (11)	0.0109 (11)	0.0070 (11)
O4	0.0371 (14)	0.0361 (14)	0.0425 (14)	0.0086 (11)	0.0105 (11)	0.0036 (11)
O6	0.0489 (16)	0.0493 (15)	0.0346 (14)	−0.0048 (12)	−0.0022 (11)	0.0123 (11)
N1	0.0307 (16)	0.0346 (16)	0.0281 (15)	0.0018 (13)	0.0001 (13)	0.0027 (12)
N4	0.0346 (16)	0.0354 (16)	0.0305 (15)	0.0030 (13)	0.0040 (12)	−0.0005 (12)
C2	0.037 (2)	0.0283 (18)	0.0314 (18)	−0.0022 (15)	0.0047 (15)	0.0008 (14)
C3	0.0315 (18)	0.0288 (18)	0.0301 (17)	−0.0003 (14)	0.0031 (15)	0.0009 (14)
C4	0.0330 (19)	0.033 (2)	0.0284 (18)	0.0013 (16)	0.0000 (14)	−0.0007 (15)
C5	0.033 (2)	0.039 (2)	0.0341 (19)	0.0052 (16)	0.0058 (15)	0.0046 (16)
C6	0.039 (2)	0.0301 (18)	0.0306 (18)	0.0007 (15)	0.0037 (16)	−0.0001 (14)
C12	0.036 (2)	0.060 (3)	0.069 (3)	−0.002 (2)	0.0122 (19)	0.013 (2)
C13	0.036 (2)	0.0353 (19)	0.0354 (18)	0.0010 (15)	0.0039 (15)	0.0018 (16)
C16	0.060 (3)	0.058 (2)	0.042 (2)	−0.006 (2)	0.0018 (19)	0.0152 (19)
C21	0.0304 (19)	0.0284 (18)	0.0288 (17)	−0.0007 (15)	0.0001 (14)	0.0028 (14)
C22	0.0282 (19)	0.0326 (19)	0.041 (2)	−0.0019 (15)	0.0017 (16)	0.0013 (16)
C23	0.038 (2)	0.0306 (18)	0.038 (2)	0.0013 (17)	0.0023 (16)	0.0003 (15)
C24	0.030 (2)	0.036 (2)	0.0353 (19)	0.0026 (16)	0.0013 (15)	0.0091 (16)
C25	0.033 (2)	0.034 (2)	0.043 (2)	−0.0048 (16)	0.0005 (16)	0.0077 (16)
C26	0.040 (2)	0.0319 (19)	0.0353 (19)	−0.0023 (16)	−0.0020 (16)	0.0001 (15)
C31	0.0357 (19)	0.037 (2)	0.0297 (18)	−0.0054 (17)	0.0040 (15)	0.0056 (16)
C32	0.042 (2)	0.053 (2)	0.035 (2)	−0.0030 (18)	0.0065 (17)	−0.0007 (18)
C33	0.059 (3)	0.071 (3)	0.036 (2)	−0.017 (2)	0.009 (2)	−0.007 (2)
C34	0.058 (3)	0.071 (3)	0.042 (2)	−0.025 (2)	−0.010 (2)	0.014 (2)
C35	0.050 (2)	0.050 (2)	0.061 (3)	−0.005 (2)	−0.014 (2)	0.021 (2)
C36	0.047 (2)	0.039 (2)	0.044 (2)	−0.0003 (18)	0.0009 (18)	0.0073 (17)
C61	0.0350 (19)	0.0334 (19)	0.0286 (18)	0.0013 (16)	0.0039 (15)	−0.0022 (14)
C62	0.041 (2)	0.049 (2)	0.034 (2)	−0.0036 (18)	−0.0036 (16)	0.0096 (17)
C63	0.042 (2)	0.044 (2)	0.038 (2)	−0.0094 (17)	0.0002 (17)	0.0078 (17)
C64	0.042 (2)	0.036 (2)	0.0270 (19)	0.0054 (16)	0.0016 (16)	0.0046 (15)
C65	0.045 (2)	0.046 (2)	0.035 (2)	−0.0027 (18)	−0.0090 (17)	0.0028 (17)
C66	0.041 (2)	0.036 (2)	0.036 (2)	−0.0047 (16)	−0.0010 (16)	0.0016 (16)

### Geometric parameters (Å, °)

**Table:**

O2—C12	1.428 (4)	C61—C62	1.384 (5)
O2—C24	1.368 (4)	C62—C63	1.395 (5)
O4—N4	1.419 (3)	C63—C64	1.380 (4)
O6—C16	1.420 (4)	C64—C65	1.381 (5)
O6—C64	1.371 (4)	C65—C66	1.379 (5)
O4—H4	0.8400	C2—H2	1.0000
N1—C6	1.489 (4)	C3—H3	1.0000
N1—C2	1.486 (4)	C5—H5A	0.9900
N4—C4	1.284 (4)	C5—H5B	0.9900
N1—H1	0.97 (3)	C6—H6	1.0000
C2—C3	1.544 (4)	C12—H12A	0.9800
C2—C21	1.510 (4)	C12—H12B	0.9800
C3—C13	1.538 (5)	C12—H12C	0.9800
C3—C4	1.505 (4)	C13—H13A	0.9900
C4—C5	1.503 (5)	C13—H13B	0.9900
C5—C6	1.529 (4)	C16—H16A	0.9800
C6—C61	1.518 (5)	C16—H16B	0.9800
C13—C31	1.516 (4)	C16—H16C	0.9800
C21—C22	1.392 (5)	C22—H22	0.9500
C21—C26	1.383 (4)	C23—H23	0.9500
C22—C23	1.377 (4)	C25—H25	0.9500
C23—C24	1.398 (4)	C26—H26	0.9500
C24—C25	1.387 (5)	C32—H32	0.9500
C25—C26	1.391 (4)	C33—H33	0.9500
C31—C36	1.395 (5)	C34—H34	0.9500
C31—C32	1.391 (5)	C35—H35	0.9500
C32—C33	1.383 (5)	C36—H36	0.9500
C33—C34	1.372 (6)	C62—H62	0.9500
C34—C35	1.368 (6)	C63—H63	0.9500
C35—C36	1.387 (5)	C65—H65	0.9500
C61—C66	1.398 (4)	C66—H66	0.9500
			
O2···C13^i^	3.251 (4)	H3···C32	2.8700
O4···N1^ii^	2.818 (3)	H3···H22	2.4300
O4···C65^iii^	3.334 (4)	H4···N1^ii^	2.0100
O2···H13B^i^	2.8700	H4···C6^ii^	2.9100
O2···H33^iv^	2.6100	H4···C22^ii^	3.0400
O2···H13A^i^	2.8700	H4···H1^ii^	2.2200
O4···H65^iii^	2.4300	H4···H22^ii^	2.4600
O4···H5A	2.2700	H5A···O4	2.2700
O6···H26^iii^	2.8100	H5A···C62	2.8900
O6···H1^v^	2.52 (3)	H5A···H62	2.4600
N1···O4^vi^	2.818 (3)	H5A···H65^iii^	2.5000
N4···C31	3.266 (4)	H5B···C62	2.8800
N4···C36	3.307 (4)	H5B···H62	2.3200
N1···H4^vi^	2.0100	H5B···N4^vi^	2.9200
N1···H22	2.8700	H6···H2	2.3400
N4···H13B	2.4400	H6···H66	2.5500
N4···H36	2.8700	H6···C63^ii^	3.0600
N4···H5B^ii^	2.9200	H12A···C32^vii^	2.9900
C12···C36^vii^	3.583 (5)	H12A···C33^vii^	2.9400
C12···C31^vii^	3.565 (5)	H12A···C36^i^	2.7600
C12···C32^vii^	3.551 (4)	H12A···H36^i^	2.5400
C12···C36^i^	3.547 (5)	H12B···C25	2.7700
C12···C33^vii^	3.580 (5)	H12B···C34^vii^	3.0700
C13···O2^viii^	3.251 (4)	H12B···C35^vii^	2.8800
C31···C12^ix^	3.565 (5)	H12B···C36^vii^	3.0100
C31···N4	3.266 (4)	H12B···H25	2.3700
C32···C12^ix^	3.551 (4)	H12C···C4^vii^	2.9100
C33···C12^ix^	3.580 (5)	H12C···C25	2.7200
C36···C12^ix^	3.583 (5)	H12C···H25	2.2500
C36···C12^viii^	3.547 (5)	H13A···C21	2.5500
C36···N4	3.307 (4)	H13A···C26	2.9200
C65···O4^x^	3.334 (4)	H13A···H32	2.5500
C65···C66^v^	3.532 (5)	H13A···H34^iv^	2.6000
C65···C65^v^	3.416 (5)	H13A···O2^viii^	2.8700
C66···C65^v^	3.532 (5)	H13B···N4	2.4400
C3···H22	3.0000	H13B···H36	2.4400
C4···H12C^ix^	2.9100	H13B···O2^viii^	2.8700
C5···H62	2.5900	H13B···C12^viii^	3.0400
C6···H4^vi^	2.9100	H16A···C21^v^	2.9100
C12···H33^iv^	3.0800	H16A···C25^v^	2.8200
C12···H13B^i^	3.0400	H16A···C26^v^	2.7000
C12···H25	2.5200	H16B···C63	2.7900
C16···H26^iii^	3.0600	H16B···H63	2.4100
C16···H63	2.5200	H16B···H2^iii^	2.5200
C21···H13A	2.5500	H16C···C63	2.7000
C21···H16A^v^	2.9100	H16C···H63	2.2100
C22···H3	2.8200	H16C···C65^vi^	2.9000
C22···H4^vi^	3.0400	H16C···C66^vi^	2.8000
C24···H33^iv^	2.7700	H22···N1	2.8700
C25···H16A^v^	2.8200	H22···C3	3.0000
C25···H12C	2.7200	H22···H3	2.4300
C25···H12B	2.7700	H22···H4^vi^	2.4600
C26···H13A	2.9200	H23···C34^xii^	3.0900
C26···H16A^v^	2.7000	H25···C12	2.5200
C32···H34^iv^	3.1000	H25···H12B	2.3700
C32···H3	2.8700	H25···H12C	2.2500
C32···H12A^ix^	2.9900	H26···H2	2.3200
C33···H12A^ix^	2.9400	H26···O6^x^	2.8100
C34···H12B^ix^	3.0700	H26···C16^x^	3.0600
C34···H23^xi^	3.0900	H32···H13A	2.5500
C35···H12B^ix^	2.8800	H33···O2^xiii^	2.6100
C36···H12B^ix^	3.0100	H33···C12^xiii^	3.0800
C36···H12A^viii^	2.7600	H33···C24^xiii^	2.7700
C62···H5B	2.8800	H34···C32^xiii^	3.1000
C62···H5A	2.8900	H34···H13A^xiii^	2.6000
C63···H16B	2.7900	H36···N4	2.8700
C63···H6^vi^	3.0600	H36···H13B	2.4400
C63···H16C	2.7000	H36···H12A^viii^	2.5400
C65···H16C^ii^	2.9000	H62···C5	2.5900
C66···H1	2.77 (3)	H62···H5A	2.4600
C66···H16C^ii^	2.8000	H62···H5B	2.3200
H1···C66	2.77 (3)	H63···C16	2.5200
H1···H66	2.4800	H63···H16B	2.4100
H1···O6^v^	2.52 (3)	H63···H16C	2.2100
H1···H4^vi^	2.2200	H65···O4^x^	2.4300
H2···H6	2.3400	H65···H5A^x^	2.5000
H2···H26	2.3200	H66···H1	2.4800
H2···H16B^x^	2.5200	H66···H6	2.5500
H3···C22	2.8200		
			
C12—O2—C24	116.8 (2)	C4—C3—H3	107.00
C16—O6—C64	117.1 (2)	C13—C3—H3	107.00
N4—O4—H4	109.00	C4—C5—H5A	110.00
C2—N1—C6	111.7 (2)	C4—C5—H5B	110.00
O4—N4—C4	111.9 (2)	C6—C5—H5A	110.00
C6—N1—H1	111.1 (18)	C6—C5—H5B	110.00
C2—N1—H1	106.3 (17)	H5A—C5—H5B	108.00
N1—C2—C3	109.7 (3)	N1—C6—H6	108.00
N1—C2—C21	109.2 (3)	C5—C6—H6	108.00
C3—C2—C21	112.9 (2)	C61—C6—H6	108.00
C2—C3—C4	108.7 (2)	O2—C12—H12A	110.00
C2—C3—C13	111.5 (3)	O2—C12—H12B	109.00
C4—C3—C13	114.1 (3)	O2—C12—H12C	109.00
N4—C4—C5	126.2 (3)	H12A—C12—H12B	109.00
N4—C4—C3	118.0 (3)	H12A—C12—H12C	109.00
C3—C4—C5	115.6 (3)	H12B—C12—H12C	109.00
C4—C5—C6	107.4 (3)	C3—C13—H13A	109.00
N1—C6—C5	107.3 (2)	C3—C13—H13B	109.00
C5—C6—C61	115.7 (3)	C31—C13—H13A	109.00
N1—C6—C61	110.4 (3)	C31—C13—H13B	109.00
C3—C13—C31	114.3 (3)	H13A—C13—H13B	108.00
C22—C21—C26	118.0 (3)	O6—C16—H16A	109.00
C2—C21—C26	121.0 (3)	O6—C16—H16B	109.00
C2—C21—C22	121.0 (3)	O6—C16—H16C	109.00
C21—C22—C23	121.2 (3)	H16A—C16—H16B	109.00
C22—C23—C24	120.1 (3)	H16A—C16—H16C	110.00
O2—C24—C23	115.3 (3)	H16B—C16—H16C	109.00
O2—C24—C25	125.0 (3)	C21—C22—H22	119.00
C23—C24—C25	119.7 (3)	C23—C22—H22	119.00
C24—C25—C26	119.0 (3)	C22—C23—H23	120.00
C21—C26—C25	122.1 (3)	C24—C23—H23	120.00
C13—C31—C32	119.1 (3)	C24—C25—H25	121.00
C13—C31—C36	122.8 (3)	C26—C25—H25	121.00
C32—C31—C36	118.1 (3)	C21—C26—H26	119.00
C31—C32—C33	120.5 (3)	C25—C26—H26	119.00
C32—C33—C34	120.5 (4)	C31—C32—H32	120.00
C33—C34—C35	120.1 (4)	C33—C32—H32	120.00
C34—C35—C36	120.0 (3)	C32—C33—H33	120.00
C31—C36—C35	120.8 (3)	C34—C33—H33	120.00
C6—C61—C62	123.1 (3)	C33—C34—H34	120.00
C6—C61—C66	119.3 (3)	C35—C34—H34	120.00
C62—C61—C66	117.6 (3)	C34—C35—H35	120.00
C61—C62—C63	121.5 (3)	C36—C35—H35	120.00
C62—C63—C64	119.8 (3)	C31—C36—H36	120.00
O6—C64—C65	115.6 (3)	C35—C36—H36	120.00
O6—C64—C63	124.8 (3)	C61—C62—H62	119.00
C63—C64—C65	119.6 (3)	C63—C62—H62	119.00
C64—C65—C66	120.4 (3)	C62—C63—H63	120.00
C61—C66—C65	121.2 (3)	C64—C63—H63	120.00
N1—C2—H2	108.00	C64—C65—H65	120.00
C3—C2—H2	108.00	C66—C65—H65	120.00
C21—C2—H2	108.00	C61—C66—H66	119.00
C2—C3—H3	107.00	C65—C66—H66	119.00
			
C12—O2—C24—C23	−174.7 (3)	C5—C6—C61—C66	168.6 (3)
C12—O2—C24—C25	4.3 (4)	C3—C13—C31—C32	74.9 (4)
C16—O6—C64—C63	8.4 (4)	C3—C13—C31—C36	−106.8 (4)
C16—O6—C64—C65	−171.6 (3)	C2—C21—C22—C23	−177.6 (3)
C6—N1—C2—C3	61.5 (3)	C26—C21—C22—C23	1.2 (5)
C6—N1—C2—C21	−174.2 (2)	C2—C21—C26—C25	177.7 (3)
C2—N1—C6—C5	−65.7 (3)	C22—C21—C26—C25	−1.0 (5)
C2—N1—C6—C61	167.5 (2)	C21—C22—C23—C24	−0.2 (5)
O4—N4—C4—C3	179.1 (2)	C22—C23—C24—O2	178.3 (3)
O4—N4—C4—C5	5.6 (4)	C22—C23—C24—C25	−0.8 (5)
N1—C2—C3—C4	−51.9 (3)	O2—C24—C25—C26	−178.1 (3)
N1—C2—C3—C13	−178.5 (2)	C23—C24—C25—C26	0.9 (5)
C21—C2—C3—C4	−173.9 (3)	C24—C25—C26—C21	0.0 (5)
C21—C2—C3—C13	59.5 (4)	C13—C31—C32—C33	177.9 (3)
N1—C2—C21—C22	−61.0 (4)	C36—C31—C32—C33	−0.4 (5)
N1—C2—C21—C26	120.4 (3)	C13—C31—C36—C35	−176.9 (3)
C3—C2—C21—C22	61.4 (4)	C32—C31—C36—C35	1.4 (5)
C3—C2—C21—C26	−117.3 (3)	C31—C32—C33—C34	−0.7 (6)
C2—C3—C4—N4	−121.5 (3)	C32—C33—C34—C35	1.0 (6)
C2—C3—C4—C5	52.7 (4)	C33—C34—C35—C36	−0.1 (6)
C13—C3—C4—N4	3.6 (4)	C34—C35—C36—C31	−1.1 (5)
C13—C3—C4—C5	177.8 (3)	C6—C61—C62—C63	178.1 (3)
C2—C3—C13—C31	−155.9 (3)	C66—C61—C62—C63	−0.4 (5)
C4—C3—C13—C31	80.5 (3)	C6—C61—C66—C65	−179.1 (3)
N4—C4—C5—C6	116.3 (3)	C62—C61—C66—C65	−0.6 (5)
C3—C4—C5—C6	−57.3 (3)	C61—C62—C63—C64	0.8 (5)
C4—C5—C6—N1	60.3 (3)	C62—C63—C64—O6	179.8 (3)
C4—C5—C6—C61	−176.1 (3)	C62—C63—C64—C65	−0.2 (5)
N1—C6—C61—C62	112.1 (3)	O6—C64—C65—C66	179.3 (3)
N1—C6—C61—C66	−69.4 (4)	C63—C64—C65—C66	−0.8 (5)
C5—C6—C61—C62	−9.9 (5)	C64—C65—C66—C61	1.2 (5)

Symmetry codes: (i) −*x*+1/2, *y*+1/2, *z*; (ii) −*x*+3/2, *y*−1/2, *z*; (iii) *x*+1/2, −*y*+1/2, −*z*; (iv) *x*−1/2, *y*, −*z*+1/2; (v) −*x*+1, −*y*+1, −*z*; (vi) −*x*+3/2, *y*+1/2, *z*; (vii) *x*−1, *y*, *z*; (viii) −*x*+1/2, *y*−1/2, *z*; (ix) *x*+1, *y*, *z*; (x) *x*−1/2, −*y*+1/2, −*z*; (xi) −*x*+1, *y*−1/2, −*z*+1/2; (xii) −*x*+1, *y*+1/2, −*z*+1/2; (xiii) *x*+1/2, *y*, −*z*+1/2.

### Hydrogen-bond geometry (Å, °)

**Table:**

*D*—H···*A*	*D*—H	H···*A*	*D*···*A*	*D*—H···*A*
N1—H1···O6^v^	0.97 (3)	2.52 (3)	3.348 (3)	144 (2)
O4—H4···N1^ii^	0.84	2.01	2.818 (3)	160
C5—H5A···O4	0.99	2.27	2.705 (4)	105
C65—H65···O4^x^	0.95	2.43	3.334 (4)	158
C12—H12B···Cg1^vii^	0.98	2.89	3.314 (4)	107
C16—H16A···Cg2^v^	0.98	2.65	3.598 (4)	162

Symmetry codes: (v) −*x*+1, −*y*+1, −*z*; (ii) −*x*+3/2, *y*−1/2, *z*; (x) *x*−1/2, −*y*+1/2, −*z*; (vii) *x*−1, *y*, *z*.
